# Different serotypes of *Escherichia coli* flagellin exert identical adjuvant effects

**DOI:** 10.1186/s12917-022-03412-3

**Published:** 2022-08-12

**Authors:** Shengmei Pang, Wenwen Wu, Qinfang Liu, Guoqiang Zhu, Qiangde Duan

**Affiliations:** 1 College of Veterinary Medicine (Institute of Comparative Medicine), Yangzhou University, 12 East Wenhui Road, Yangzhou, 225009 China; 2 Jiangsu Co-Innovation Center for Prevention and Control of Important Animal Infectious Diseases and Zoonoses, Joint International Research Laboratory of Agriculture and Agri-Product Safety of Ministry of Education of China, Yangzhou, China; 3 Jiangsu Joint Laboratory for International Cooperation in Prevention and Control of Important Animal Infectious Diseases and Zoonoses, Yangzhou, 225009 China; 4 Department of Anatomy and Physiology, Kansas State University College of Veterinary Medicine, Manhattan, KS USA

**Keywords:** Flagellin, TLR5, Adjuvanticity, Serotype, *E. coli*

## Abstract

Bacterial flagellin is a potent powerful adjuvant, which exerts its adjuvant activity by activating the Toll-like receptor 5 (TLR5) signaling pathway to induce host pro-inflammatory responses. Flagellin of *Salmonella typhimurium* (*S. typhimurium*) has shown strong adjuvant effects for a variety of vaccine candidates, however, the adjuvanticity of different serotypes of *Escherichia coli* (*E. coli*) flagellin (FliC) is unclear. To explore the adjuvant activity of different serotypes of *E. coli* flagellin, FliC_H1_, FliC_H7_, and FliC_H19_ recombinant flagellins were prokaryotically-expressed and purified. The adjuvanticity of three recombinant flagellins was evaluated by analyzing their abilities to induce the IL-8 production in human colorectal adenocarcinoma (Caco-2) cells and the immune responses to co-administrated FaeG antigen in mice. Sequence analysis showed that the N-and C-terminal regions are highly conserved, whereas the central region is hypervariable. The TLR5 recognized site is identical among these three serotypes of flagellins. Coomassie blue staining SDS-PAGE showed the molecular mass of FliC_H1_, FliC_H7_, and FliC_H19_ recombinant flagellin are 66 kDa, 64 kDa, and 68 kDa, which can be recognized by anti-FliC_H1_, FliC_H7_, and FliC_H19_ serum, respectively. Moreover, the flagellin serotypes induced similar levels of IL-8 and TNF-α production in Caco-2 cells, anti-FaeG specific IgG antibodies in mice, and IL-4 production in mice spleen cells. Our results indicated that *E. coli* flagellins can be an adjuvant for vaccine candidates and that different serotypes of *E. coli* flagellins possess identical adjuvant effects.

**Supplementary Information:**

The online version contains supplementary material available at 10.1186/s12917-022-03412-3.

## Background

The bacterial flagellum is well known as a locomotive organelle responsible for movement and chemotaxis, which is commonly expressed in both Gram-positive and Gram-negative bacteria. Moreover, flagellum contributes to bacterial pathogenesis by promoting bacterial adherence, colonization, invasion, biofilm formation, and host pro-inflammatory responses [1–3]. The bacterial flagellum is a complex apparatus, more than 50 genes are required for flagellar assembly, structure, and function. A flagellum consists of three parts: the basal body, the hook, and the filament. The basal body acts as a rotary motor, and the hook connects the motor and filament. The filament functions as a propeller, which is built by ~ 20,000 flagellin monomers. Flagellin, the main structural protein monomer, is encoded by the *fliC* gene in *Escherichia coli* (*E. coli*). Sequence analysis of *fliC* genes revealed that the N- and C-terminals are highly conserved among bacterial species, whereas the central region undergoes high variation in different species and serotypes.

A study on the crystallographic structure of *Salmonella typhimurium* (*S. typhimurium*) flagellin illustrated that it consists of four domains (D0, D1, D2, and D3). In the amino acid sequence, the domains of flagellin are arranged as D0-D1-D2-D3-D2-D1-D0 from the N-terminus to C-terminal. The highly conserved D0 and D1domains formed by an α-helix structure are located in the core of the flagellin, which hinders the activation of TLR5 during the assembly of flagellin into flagella filaments. By contrast, the hypervariable D2 and D3 domains mostly made up of β-stands are exposed on the surface of the filament, which is essential for the immunogenicity of flagellin [4, 5]. Different domains are associated with different functions. The highly conserved D0 and D1 domains are responsible for secretion and polymerization of flagellin and are required for activating the host’s innate immune responses to exert its adjuvant effect [6, 7]. The hypervariable D2 and D3 domains are involved in the different H-serotypes and the immunogenicity of flagellin [8]. The immunogenicity is based upon the ability of flagellin to induce anti-flagellin-specific antibodies. Thus far, a total of 53 different H serotypes have been recognized, based on considerable variability of *E. coli* flagellin in ultrastructure, which is an important antigen to subtype *E. coli* in epidemiological studies [9].

As a highly evolutionarily conserved, pathogen-associated molecular pattern (PAMPs), flagellin is recognized by the pattern recognition receptors (PRRs), as well as by B-cell and T-cell receptors. Recognition triggers the host’s innate immune system and proper adaptive immunity, contributing to the immediate clearance of pathogens from the host. Therefore, flagellin has been identified as a unique and highly efficient activator of immune responses [10]. Two types of flagellin receptors have been identified: (1) the cell surface-localized Toll-like receptor 5 (TLR5) and (2) the cytosolic NOD-like receptor protein 4 (NLRC4) inflammasome receptor NAIP5/6 [11]. Bacterial flagellin induces immune responses via binding to and activating these receptors. In *S. typhimurium* flagellin, the key site recognized by TLR5 is located in the N-terminal 89–96 amino acid residues (QRVRELAV) of its D1 domain, whereas NAIP5/6 recognizes flagellin in the C-terminal 35aa leucine-rich helical region [12–14]. Once bound to extracellular TLR5, flagellin activates the MyD88-dependent signaling pathway and elicits pro-inflammatory cytokine production mediated by NF-kB and MAPK signaling pathways [15–17]. *Salmonella* flagellin also enhances IL-8 secretion in both HeLa cells and polarized T84 cell monolayers that express TLR5. Therefore, the binding with TLR5 is not only required for inducing pro-inflammatory cytokine secretion but also enables flagellin to boost the immune response against co-administered antigens as an adjuvant [18]. In the cytosol, the interaction of flagellin and the NAIP5/6 receptor enhances the secretion of IL-18 and IL-1β and leads to cell death [19]. Because of its ability to effectively stimulate the host immune system and strengthen the immunogenicity of co-administrated antigens, the adjuvant activity of *S. typhimurium* flagellin has been extensively studied in a variety of animal models with different routes and bacterial, viral, fungal, and parasitic antigens [20–22].

The structures and adjuvant properties of the *S. typhimurium* flagellin have been extensively studied, however, the adjuvant properties of different serotypes of *E. coli* flagellin are poorly understood. In theory, it is more convincing to study the immune adjuvant effect of different serotypes of flagellin including all 53 serotypes; however, it is unrealistic to include all 53. Therefore, we selected three existing serotypes in the laboratory: (1) FliC_H1_ commonly expressed on Shiga toxin-producing *Escherichia coli* (STEC) strains that causes edema disease; (2) FliC_H7_ expressed on the enterohemorrhagic *Escherichia coli* (EHEC) strains that causes water diarrhea and hemolytic-uremic syndrome; (3) FliC_H19_ commonly expressed on the enterotoxigenic *Escherichia coli* (ETEC) strains associated with piglet diarrhea. These three were representatives to explore the immunoadjuvant activities of flagellin in different serotypes of *Escherichia coli*. We expressed and purified three different serotypes of *E. coli* recombinant flagellin (FliC_H1_, FliC_H7_, and FliC_H19_) and further evaluated their adjuvant effects via comparing the TLR5 bioactivities in vitro in a human Caco-2 cell line, the specific antibody responses against the model FaeG antigen after co-immunized in mice, and the expression level of pro-inflammatory cytokines in spleen cells of immunized mice.

## Results

### Characterization of *fliC*_H1_, *fliC*_H7_, and *fliC*_H19_ genes

**Fig. 1:**
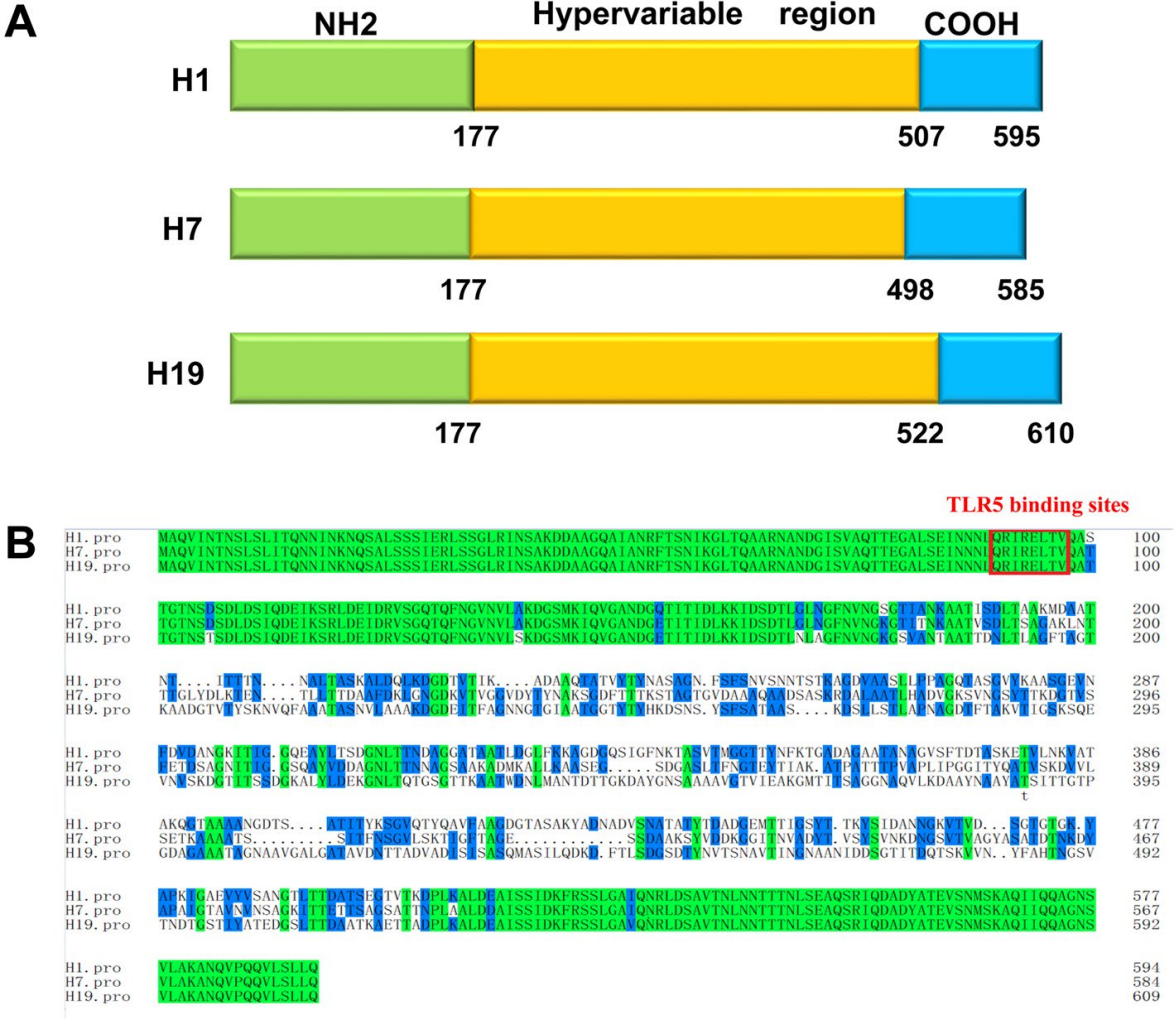
Characterization of the sequences of FliC_H1_, FliC_H7_, and FliC_H19_ flagellin. **A** Schematic representation of the FliC_H1_, FliC_H7_ and FliC_H19_ genes. The sequences in N-terminal regions (green) and C-terminal regions (blue) are highly conserved, whereas the sequences in central regions are hypervariable (orange). **B** Amino acid alignment of FliC_H1_, FliC_H7_, and FliC_H19_ sequences was performed by the MegAlign program. The amino acid residues that were the same among the three serotypes of *E. coli* flagellins are in green. The TLR5 recognized site (QRIRELTV) of these three serotypes of FliC was located in the 90–97^th^ amino acid of the N-terminal, circled in a red rectangle

Analysis of *E. coli* flagellin genes showed that the length of the coding sequence of *fliC*_H1_, *fliC*_H7_, and *fliC*_H19_ genes is 1788, 1758, and 1842 bp, which codes for 595, 585, and 610 amino acid residues, respectively (Fig. 1A). An alignment of the three serotypes of flagellin discovered that the N- and C-terminal regions are highly conserved with a concentrated variability in length and amino acid content in the central region, accounting for the H-serotype specificity. Protein sequences of the 177 amino acids in the N-terminal and the 88 amino acids in the C-terminal have high similarity (> 96%) among the three *fliC*, whereas the sequence homologies in central hypervariable regions containing several gaps are less than 50%. The TLR5 recognized site (QRIRELTV), located in the 89–96^th^ amino acid of the N-terminal, is identical to the three serotypes of flagellin (Fig. 1B).

### Expression and purification of recombinant FliC proteins

The recombinant FliC proteins were expressed in *E. coli*, then extracted and purified.

**Fig. 2:**
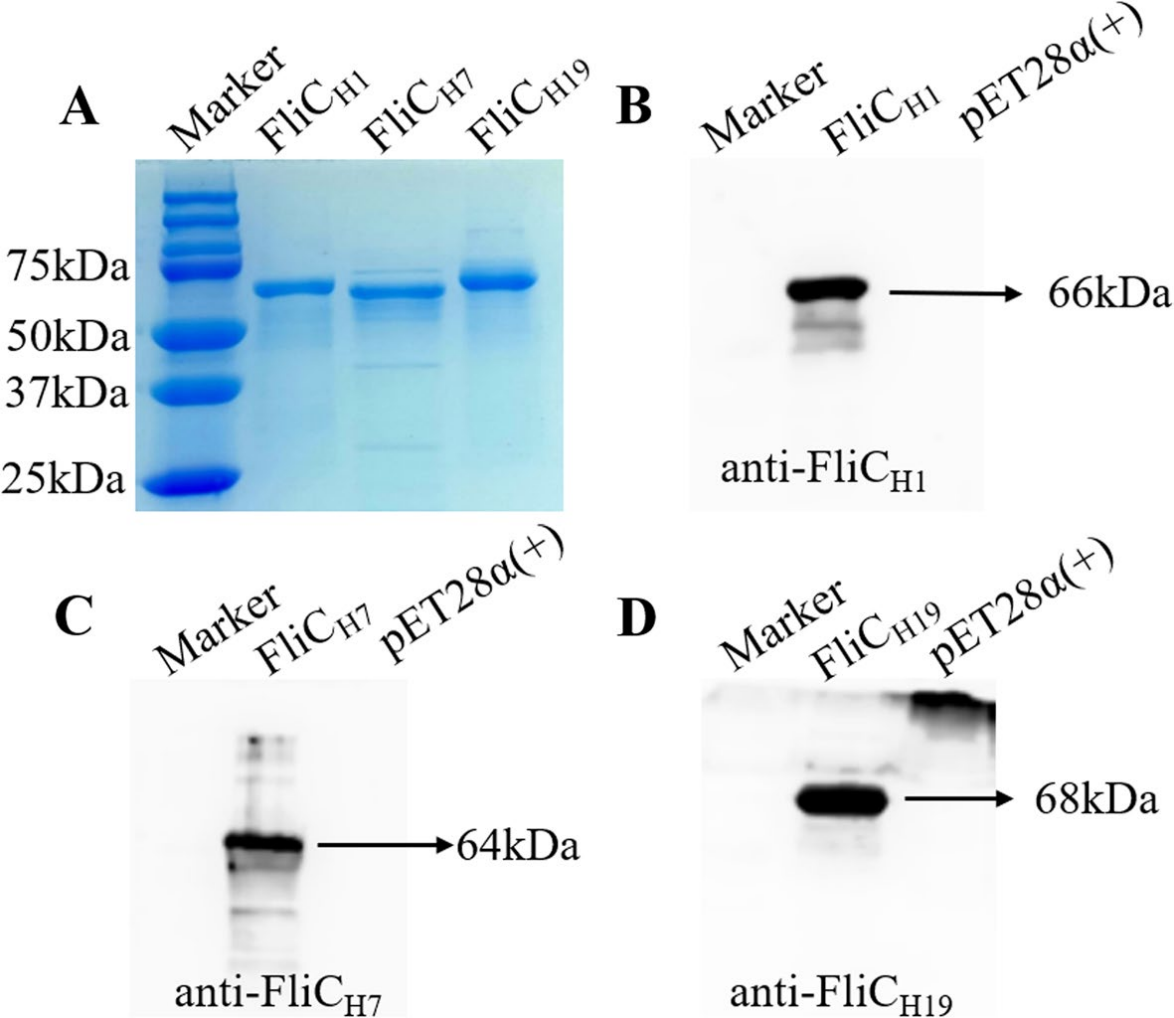
Extraction and detection of recombinant FliC_H1_, FliC_H7_, and FliC_H19_ flagellin proteins. **A** Coomassie blue staining of recombinant FliC_H1_, FliC_H7_, and FliC_H19_ proteins in SDS-PAGE. **B**–**D** The expressed FliC_H1_, FliC_H7_, and FliC_H19_ flagellin proteins were detected using rabbit anti-FliC_H1_, FliC_H7_, and FliC_H19_ (1:10,000) antiserum, respectively. HRP-labeled goat anti-rabbit IgG (1:20,000; Sigma) was used as the secondary antibodies in western blotting. Purified His_6_-tag proteins expressed from BL21 *E. coli* harboring an empty pET28α ( +) vector served as a negative control

SDS-PAGE in conjunction with Coomassie blue staining revealed that specific bands correspond to FliC_H1_, FliC_H7_, and FliC_H19_ with molecular weights of approximately 66 kDa, 64 kDa, and 68 kDa, which are the expected sizes of these proteins (Fig. 2A). The recombinant FliC_H1_, FliC_H7_, and FliC_H19_ proteins can be recognized by anti-FliC_H1_, anti-FliC_H7_, and anti-FliC_H19_ rabbit polyclonal antibodies (TBC, China), respectively (Fig. 2B–D). This revealed that the secondary structures and antigenic reactivity of the recombinant proteins were maintained after the purification process.

### TLR5 activity of recombinant FliC proteins

**Fig. 3:**
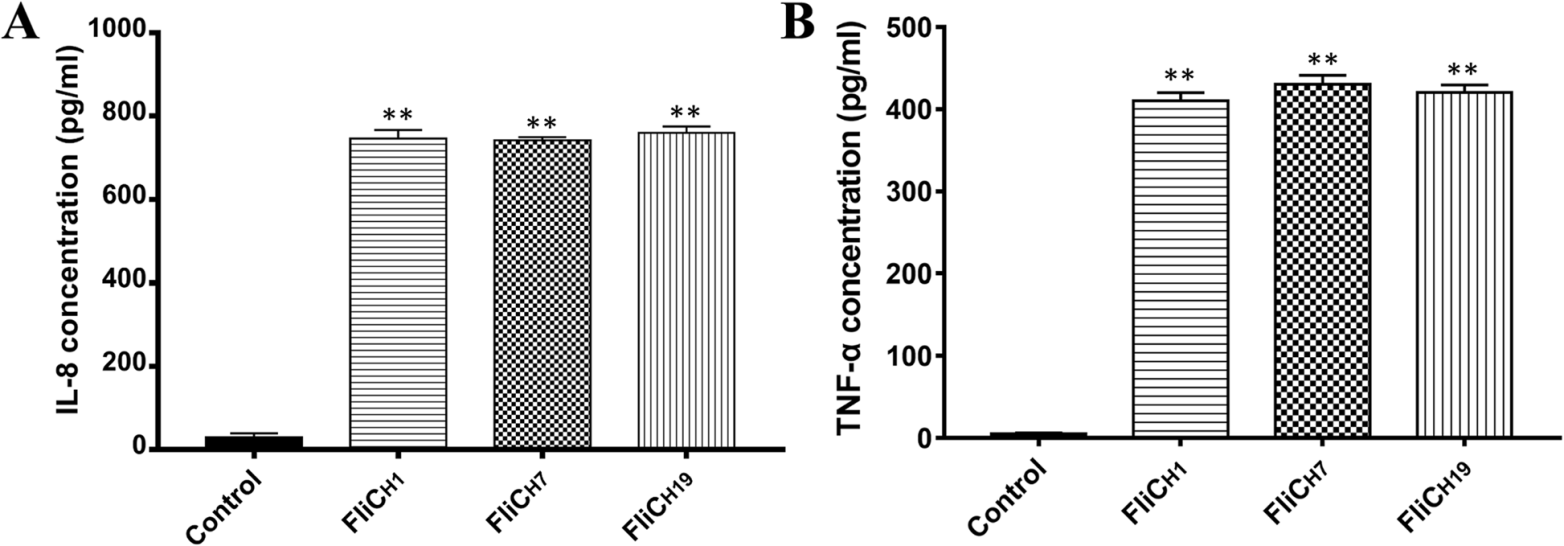
TLR5 activity of recombinant FliC_H1_, FliC_H7_ and FliC_H19_ flagellin proteins. Caco-2 cells were stimulated with 5 μg/mL purified recombinant FliC_H1_, FliC_H7,_ and FliC_H19_ flagellins for 6 h. The level of IL-8 and TNF-α secretion in the supernatant was measured by ELISA. Each sample was tested in duplicate. Bars represent the mean ± standard deviation from the three independent experiments

The TLR5-mediated signaling pathway stimulates NF-κB activation, which regulates the expression of pro-inflammatory cytokines IL-8 and TNF-α. To evaluate the TLR5 signaling activation by the recombinant FliC_H1_, FliC_H7_, and FliC_H19_ proteins, the TLR5 receptor expressed cell line, Caco-2, was used for the stimulation of three purified recombinant FliC proteins. The results showed that the levels of IL-8 and TNF-α induced by FliC_H1_, FliC_H7_, and FliC_H19_ recombinant proteins were significantly higher compared to those of the control group, however, no significant differences were observed among the three different serotypes of flagellin. This indicated that three flagellins possess strong TLR5-binding effects and relative TLR5 activation ability in vitro (Fig. 3).

### Anti-FaeG antibody titers induced by the different immunization regimes

**Fig. 4:**
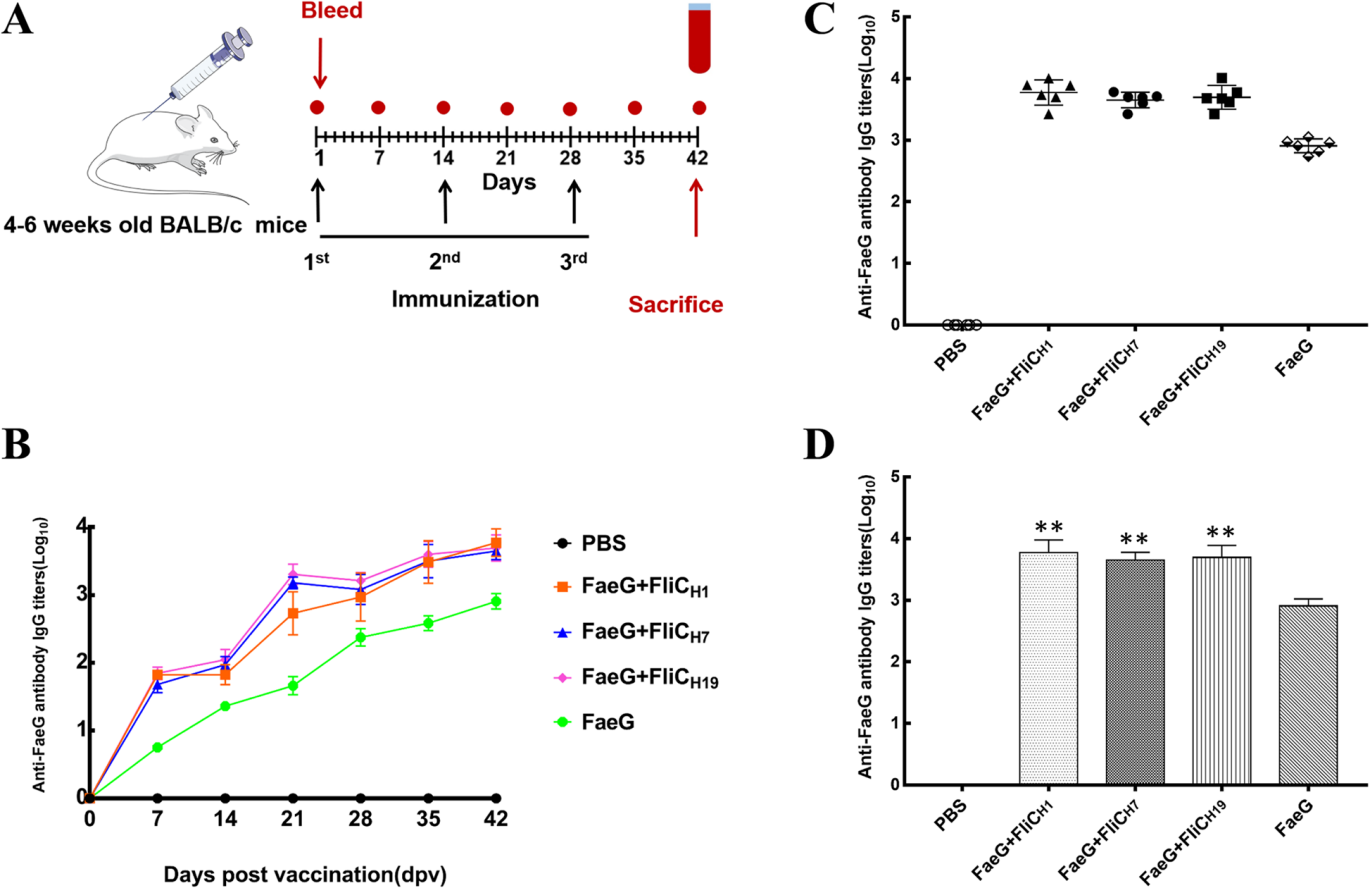
Induction of anti-FaeG IgG responses in mice. **A** Schematic diagram of the immunization program. Mice were immunized three times at bi-weekly intervals. Blood was collected from the caudal vein at 7, 14, 21, and 28 dpv (**B**) Longevity of the anti-FaeG IgG response in mice groups on different days. Data represent mean ± SD from six mice per group. **C** The final anti-FaeG total IgG antibody titers of mice serum after the third immunization. The antibody titers are calculated by multiplying the highest serum dilution that gave an OD reading > 0.3 after background subtraction and is presented in log_10_. Results are expressed as mean ± standard deviation of at least three independent experiments. ** indicate the anti-FaeG IgG antibody titers over group immunized FaeG alone (*P* < 0.01)

For this study, mice have immunized SC with FaeG admixed with purified FliC_H1_, FliC_H7_, or FliC_H19_ recombinant protein, and anti-FaeG antibodies were determined by indirect ELISA. As shown in Fig. 4B, mice immunized with the FaeG antigen supplied with FliC_H1_, FliC_H7_, or FliC_H19_ as the adjuvant exhibited a progressive increase in serum anti-FaeG IgG responses from day 7 to 42. Compared with the FaeG alone immunized group, mice vaccinated with FliC_H1_, FliC_H7_, or FliC_H19_ as an adjuvant had a significantly higher level of anti-FaeG IgG antibody titers (*P* < 0.01). However, the anti-FaeG IgG antibody titers had no significant difference among the three groups (*P* > 0.05) (Fig. 4C).

### The induction of inflammatory cytokines in spleen cells by flagellin

**Fig. 5:**
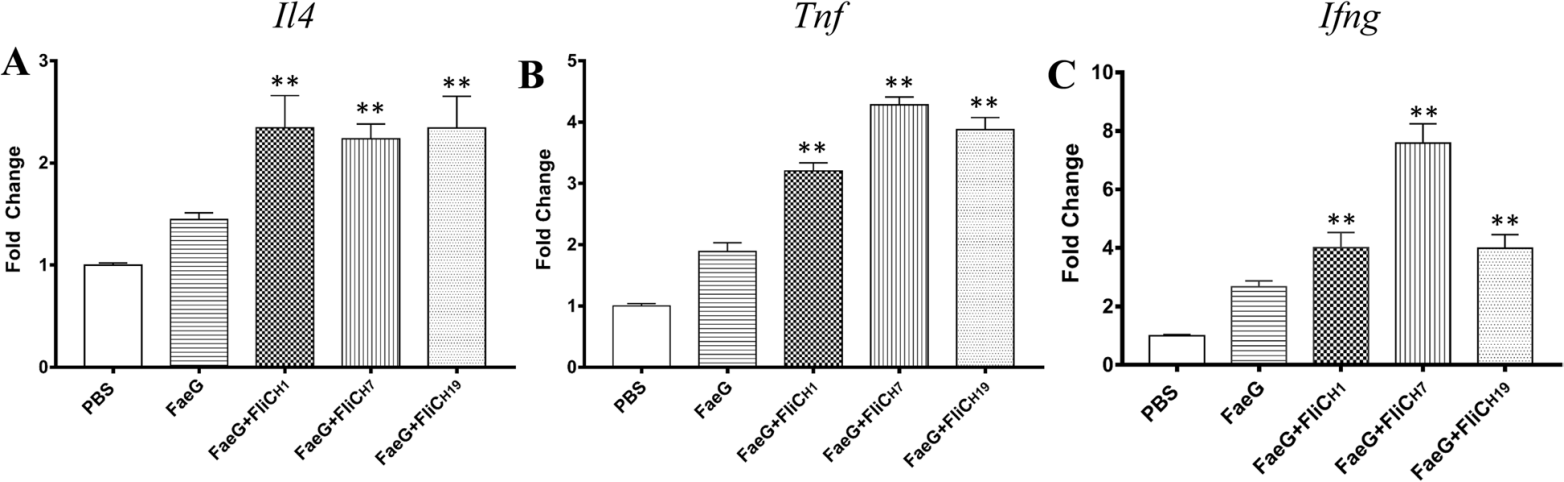
The mRNA level of pro-inflammatory cytokines in splenocytes. Splenocytes collected from the vaccinated mice were cultured in medium alone or the presence of FaeG protein for 48 h. **A** Splenocytes were analyzed with qRT-PCR for *Il4* gene expression. **B** Splenocytes were analyzed with qRT-PCR for *Tnf* gene expression. **C** Splenocytes were analyzed with qRT-PCR for *Ifng* gene expression. Double asterisks indicate statistical significance over the FaeG alone immunized group (*P* < 0.01). Results are expressed as mean ± SD from three independent experiments. Each sample was tested in triplicate

The expression of *Il4*, *Tnf*, and *Ifng* genes in spleen cells of immunized mice was measured by qRT-PCR. The results showed that the expression of *Il4*, *Tnf*, and *Ifng* was significantly higher than those in the FaeG alone immunized group (*P* < 0.01). No statistically significant differences were observed in the expression of the *Il4* gene among the three different serotypes of flagellin immunized groups (*P* > 0.05). However, a statistically significantly higher upregulation of *Tnf* was observed in FliC_H7_ than that in FliC_H1_ (*P* < 0.01) and FliC_H19_ (*P* < 0.05) at the same incubation which is consistent with the trend to *Ifng* (*P* < 0.05) (Fig. 5).

## Discussion

As an adjuvant, flagellin has several advantages compared with other adjuvants: (1) flagellin can induce both humoral and cellular immune responses in the host; (2) even in very low doses, it can exert a strong adjuvant effect; (3) its strong plastic structure permits it to insert exogenous antigen at the C-terminal, N-terminal, and hypervariable domains of the protein, but doesn’t influence its TLR5 bioactivity; (4) it has low toxicity and fewer side effects; (5) it can enhance the cross-immune protection of related antigens. Despite these advantages, there is no commercially available vaccine containing flagellin as an adjuvant, primarily because the exact mechanisms of its adjuvanticity remain unclear. In this study, to investigate the adjuvant activity of different serotypes of flagellin derived from *E. coli*, we cloned and expressed the FliC_H1_, FliC_H7_, and FliC_H19_ recombinant flagellin proteins. Our in vitro TLR5 bioactivity and in vivo adjuvanticity results indicated that the three different serotypes of flagellin (FliC_H1_, FliC_H7_, and FliC_H19_) have seemingly identical adjuvant activity.

The sequence alignment comparison showed that the amino acids in the N-(177aa) and C-terminal (88aa) are highly conserved among the three serotypes, whereas the length and amino acids in the central region are variable. Moreover, the TLR5 recognized site (QRIRELTV) of the three serotypes of flagellin located in 89–96 aa in the N-terminal is identical. In addition, the R90, E114, and Q97 residues are reported to be important for their TLR5 binding activity and account for nearly 20% of total interactions [23]. They are also 100% homologous across the three serotypes. The TLR5 recognized site is only the amino acid residue different (I–V) from *S. typhimurium* flagellin. Further studies are required to understand whether the mutation in this amino acid can cause the differences in TLR5 binding activity and adjuvanticity between these two species.

TLR5 bioactivity assay suggested that all three recombinant flagellins can elicit IL-8 secretion in Caco-2 cells that express TLR5 receptors, and the level of IL-8 is not significantly different among them. Results indicated that three different serotypes of *E. coli* flagellin possess similar TLR5 bioactivity. Hajam et al. [23] reported that co-administration of flagellin with foot-and-mouth disease virus (FMDV) antigen via an intradermal route elicited specific anti-FMDV neutralizing antibodies. In addition, using flagellin as an adjuvant and co-mixing with *mycobacterial* protein Ag85B, avian influenza H5N1 antigen, and *Plasmodium* falciparum CS protein, improves the immunogenicity of these antigens and induces specific IgG antibodies against these antigens [24]. In our study, the adjuvant properties of three different serotypes of *E. coli* recombinant flagellins were evaluated in mice SC immunized with purified FaeG protein admixed. The three flagellins induced similar secretion of IL-8 and TNF-α, indicating that the binging affinity to TLR5 was identical, which was consistent with the results of the schematic representation of the sequences of flagellins (89–96^th^ aa).

Consistent with previous studies, our results showed that mice can develop a robust immune response to the co-administrated FaeG antigen when using these flagellins as adjuvants. All the three different serotypes of *E. coli* flagellin can induce anti-FaeG specific IgG antibody production, however, the level of anti-FaeG antibody titers is not significantly different among them. Data from antibody titter and biological activity of the flagellins from two different experiments were similar. The immune response triggered by flagellin is T-cell-dependent. Flagellin stimulates the secretion of inflammatory cytokines, such as TNF-α, IFN-γ, and IL-4, to elevate the innate and specific adaptive immune response. Specifically, the results displayed a superiority of IFN-γ and TNF-α production from T cells (CD8 ^+^ T cell / CD4 ^+^ T cell) but low IL-4 (CD4 ^+^ T cell). Th1 cytokine TNF-α is a key molecule that coordinates inflammatory responses and cytokine cascade activation. Moreover, antigen-specific IFN-γis the most important for Th1-type immune responses in early defense against pathogen infection [25]. Different serotypes of *Escherichia coli* flagellin can induce a strong Th1-like response, as indicated by *Tnf* and *Ifng* expression.

## Conclusion

The results presented in this study suggested that flagellins from *E. coli* serve as a promising and attractive adjuvant for vaccine candidates. The TLR5 activity in vitro and adjuvant effect in vivo of these three serotypes are identical. As a consequence, they can function as adjuvants co-administrated with antigens against pathogens. These results enrich the knowledge on the adjuvant effect of *Escherichia coli* flagellin and provide insight into the mechanisms of flagellin as adjuvants.

## Methods

### Bacterial strains and plasmids

Shiga toxin-producing *E. coli* (STEC) F107/86 (O139:H1) [26], Enterohemorrhagic *E. coli* (EHEC) EDL933 (O157:H7) [27], and enterotoxigenic *E. coli* (ETEC) C83902 (O8:K88:H19) [28] were used to amplify the *fliC*_*H1*_, *fliC*_*H7*_, and *fliC*_*H19*_ gene, respectively. They were grown in Luria–Bertani (LB) broth or on LB agar plates at 37 °C. Expression vector pET28α ( +) (Novagen, USA) and *E. coli* strains DH5α and BL21 (DE3) (TIANGEN, China) were used for gene cloning and subsequent recombinant protein expression.

### PCR amplification and cloning of *fliC* genes

The full-length *fliC*_*H1*_, *fliC*_*H7*_*,* and *fliC*_*H19*_ genes were amplified by PCR from STEC F107/86, EHEC EDL933, and ETEC C83902 genomic DNA, using the FliC-F and FliC-R specific primers (Table 1), containing *BamH*I and *Sal*I restriction enzymes sites (underlined), at the 5’ end of each primer, respectively. The purified PCR products and the prokaryotic expression vector pET28α ( +) were digested by *BamH*I-HF and *Sal*I-HF restriction enzymes (NEB, USA). Finally, the successful construction of FliC-pET28α ( +) recombinant plasmids was confirmed by both PCR and DNA sequencing.

**Table 1:** Primers used in this study

Gene	Primer sequence(5’-3’)
*fliC*-F	CGCGGATCCATGGCACAAGTCATTAATACCAACAG
*fliC*-R	GAGCGTCGACTTAACCCTGCAGCAGAGACAGAAC
*GAPDH*-F	GCCTTCCGTGTTCCTACCC
*GAPDH*-R	TGCCTGCTTCACCACCTTC
m*Il4*-F	ACAGGAGAAGGGACGCCAT
m*Il4*-R	GAAGCCCTACAGACGAGCTCA
m*Ifng*-F	TCAAGTGGCATAGATGTGGAAGAA
m*Ifng*-R	TGGCTCTGCAGGATTTTCATG
m*Tnf*-F	AGCCCCCAGTCTGTATCCTT
m*Tnf* -R	CTCCCTTTGCAGAACTCAGG

### Sequence analysis of *fliC* genes from *E. coli*

Multiple alignments of nucleotide and amino acid sequences of FliC_H1_, FliC_H7_, and FliC_H19_ were performed using DNAStar's (Madison, WI, USA) Lasergene software MegAlign program.

### Expression and purification of recombinant flagellin proteins

Recombinant *E. coli* flagellin proteins were expressed and purified as previously described [29]. Briefly, a positive single recombinant *E. coli* colony was inoculated to LB medium containing kanamycin with 30 μg/mL and grown overnight at 37 ºC shaker (220 rpm). The next day, 5 mL of the subculture was transferred to 500 mL of fresh 2 × YT medium (2 × Yeast Extract Tryptone) supplemented with kanamycin at a final concentration of 30 μg/mL. Bacterial culture was induced by 1 mM isopropyl-β-D-1-thiogalactoside (IPTG) for an additional 4 h when the OD_600_ reached ~ 0.6. Total recombinant flagellin protein was extracted with a bacterial protein extraction reagent (B-PER) (Thermo Scientific, Rochester, NY, USA) from the harvested bacterial cells. According to the manufacturer’s instructions, extracted recombinant 6 × His tagged inclusion body proteins were purified from the total protein extract using protino Ni-TED 2000 packed columns (MACHEREY–NAGEL, Germany). Purified His_6_-tag proteins expressed from *E. coli* harboring an empty pET28α ( +) vector served as a negative control. The purified recombinant flagellin proteins were confirmed by sodium dodecyl sulfate–polyacrylamide gel electrophoresis (SDS-PAGE) with Coomassie blue staining and were quantified by a Bradford assay. Subsequently, the target protein was recognized with anti-FliC_H1_ rabbit serum (1:20,000), anti-FliC_H7_ rabbit serum (1:20,000), and anti-FliC_H19_ rabbit serum (1:20,000) in western blot images.

### Endotoxin removal and measurement

Endotoxin in FliC_H1_, FliC_H7_, and FliC_H9_ recombinant proteins was removed by Pierce™ High Capacity Endotoxin Removal Resin (Thermo Fisher Scientific). Specific steps were performed following protocol from the manufacturer. The residual LPS levels were determined by a Pierce™ Chromogenic Endotoxin Quant Kit (Thermo Fisher Scientific) to ensure that the LPS content was < 0.05 EU/ml.

### TLR5 bioactivity of flagellins assay in vitro

The human colon adenocarcinoma cell line (Caco-2) expressing the TLR5 receptor was used to measure IL-8 and TNF-α induced by purified recombinant FliC_H1_, FliC_H7_, and FliC_H19_ proteins according to a previous study [20]. Briefly, Caco-2 cells were cultured in a six-well tissue culture plate (Corning, USA) at a seeding density of 5 × 10^5^ cells for each well. When the cells reached a confluent monolayer, they were treated with 5 μg/mL purified recombinant flagellins for 6 h. The untreated cells were used as a negative control. According to the manufacturer’s instructions, the expression of IL-8 and TNF-α in the cell supernatants was tested by a human IL-8 ELISA kit and a human TNF-α ELISA kit (NeoBioscience, China). All samples were tested in duplicate.

### Immunization of mice

All animal experiments were approved by Yangzhou University Institutional Animal Care and Use Committee (202,103,034). Mice immunization complied with the National Institute of Health guidelines for the ethical use of animals in China. Mice were obtained from the Animal Experiment Center of Yangzhou University (SCXK(Su)2017–0007). A total of 30 8-week-old, specific pathogen-free (SPF) female BALB/c mice were randomly divided into five groups (*n* = 6). The FaeG recombinant protein (the major subunit of F4 fimbriae) was used as the co-administered antigen. In group 1, mice were subcutaneously (SC) immunized with 50 µg of the FaeG protein combined with 50 µg purified FliC_H1_ protein. In group 2, mice were injected subcutaneously with 50 µg of the FaeG protein mixed with 50 µg purified FliC_H7_ protein. In group 3, mice were SC immunized with 50 µg of the FaeG protein combined with 50 µg purified FliC_H19_ protein. In group 4, mice were SC immunized with 50 µg of the FaeG protein in PBS. In another group, each of the mice received an injection of 100 µl of sterile PBS as the negative control group. Each mouse received two booster injections at the same dose as the primary immunization at a 2-week interval. Blood was collected from the caudal vein at 7, 14, 21, and 28 days post-vaccination (dpv). All mice were sacrificed 2 weeks after the second booster for spleen cell harvesting to perform a splenic inflammatory cytokine assay (Fig. 4A).

### Mouse serum anti-FaeG specific IgG antibody titration assay

Antibody responses against FaeG were measured using an enzyme-linked immunosorbent assay (ELISA) as previously described [30]. Briefly, purified K88ac fimbriae were diluted in ELISA coating buffer and coated onto 96-well ELISA plates (Corning, USA) at 500 ng/well. Plates were blocked with 10% non-fat milk at 37 ℃ for 1 h and then washed three times with PBST (PBS + 0.05%, Tween20). The plates were incubated with each mouse serum sample diluted two-fold from 1:400 to 1:25,600 with PBST in 100 µL/well at 37 ℃ for 1.5 h. Horseradish peroxidase (HRP)-labeled goat anti-mouse IgG (Sigma, USA) was used as the secondary antibody, diluted in PBST (1:5000). The OD value of each well was measured at 650 nm using a microplate reader after exposure to 3, 3’, 5, 5’-tetramethylbenzidine (TMB) color development solution (Beyotime, China) at room temperature for 30 min. The anti-FaeG antibody titers are calculated by multiplying the highest serum dilution that gave an OD reading > 0.3 after background subtraction and a log_10_ transformation of the result. All experiments were conducted in triplicate.

### Pro-inflammatory cytokines measured in mice spleen cells

The primary splenic cells of mice in the FliC_H1_, FliC_H7_, FliC_H19_, FaeG, and PBS immunization groups were isolated aseptically and seeded into RPMI1640 (Gibco, USA) containing 0.1% fetal bovine serum (FBS) in a six-well plate (NEST, Shanghai, China). The spleen cells of the mice were stimulated with purified FaeG protein (50 µg/mL) for 48 h. Then, total RNA was extracted from the spleen cells using the TRNzol solution (TianGen, Beijing, China) according to the manufacturer’s instructions. Nanodrop spectrophotometry was used to measure the quality of RNA. Reverse transcription of the extracted RNA into cDNA using a FastKing gDNA Dispelling RT SuperMix kit (TianGen, Beijing, China). To determine the T cell immune responses induced by three different serotypes of flagellin, the primers of *Il4, Ifng*, and *Tnf* used for qPCR analyses are shown in Table 1. The PCR conditions were as follows: 95 ℃ for 5 min, followed by 40 cycles of 95 ℃ for 10 s, 60 ℃ for 30 s; additionally, a melting curve was performed for 95 ℃ for 15 s, 60 ℃ for 60 s, 95 ℃ for 15 s. Melting curve analysis was used to determine the specificity of the qPCR product for each pair of primers. Each sample was analyzed in triplicate for qPCR. All data were normalized to the endogenous reference gene *GAPDH* and the gene expression was calculated by the 2^−ΔΔCt^ method. The fold changes of expression of pro-inflammatory cytokines in different serotypes of *Escherichia coli* flagellin were compared.

### Statistical analysis

Data were presented as means with the standard deviation (SD) and analyzed using GraphPad Prism version 6.0 software (GraphPad Software, USA). Differences among immunized groups were calculated with a one-way analysis of variance with a confidence interval of 95%. *P* > 0.05 was considered as NS (Not significant). * *P* < 0.05 indicated a statistical difference and, ** *P* < 0.01 indicated a significant statistical difference between groups.

## Supplementary Information

**Additional file 1. **

## Data Availability

The datasets used and/or analyzed during the current study are available from the corresponding author upon reasonable request. DNA sequences data of the *E. coli* flagellin allele H1, H7, and H19 are available in GenBank repository (Accession Numbers: ON804529-ON804531).

## Abbreviations

B-PER: Bacterial protein extraction reagent
dpv: Days post vaccination
*E. coli*: *Escherichia coli*
ELISA: Enzyme-linked immunosorbent assay
FBS: Fetal bovine serum
FMDV: Foot-and-mouth disease virus
IPTG: Isopropyl-β-D-1-thiogalactoside
NLRC4: NOD-like receptor protein 4
PAMPs: Pathogen-associated molecular patterns
PRRs: Pattern recognition receptors
SDS-PAGE: Sodium dodecyl sulfate polyacrylamide gel electrophoresis
*S. **typhimurium*: *Salmonella* *typhimurium*
SPF: Specific pathogen-free
TLR5: Toll-like receptor 5
