# Supplementary figures and images for: Different serotypes of Escherichia coli flagellin exert identical adjuvant effects
Source: BMC Vet Res. 2022 Aug 12;18:308. doi: 10.1186/s12917-022-03412-3 (PMC9373361; doi:10.1186/s12917-022-03412-3)

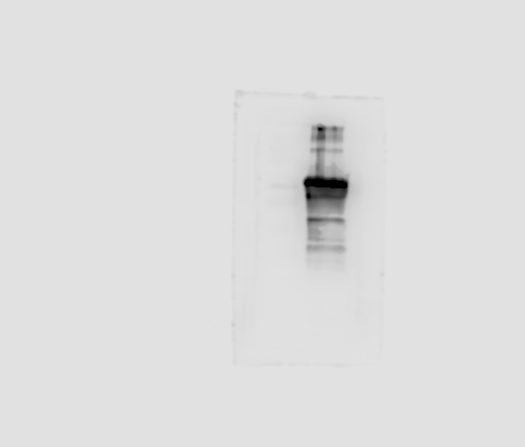

Supplement: Supplementary file 1 — Additional file 1. [file 12917_2022_3412_MOESM1_ESM.zip › 1-H7 blot original image-2.tif]

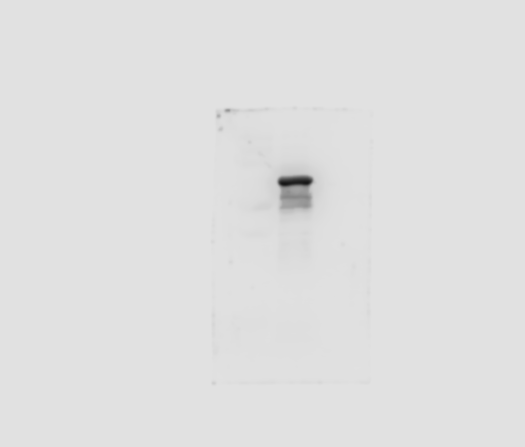

Supplement: Supplementary file 1 — Additional file 1. [file 12917_2022_3412_MOESM1_ESM.zip › H1 blot original image-1.tif]

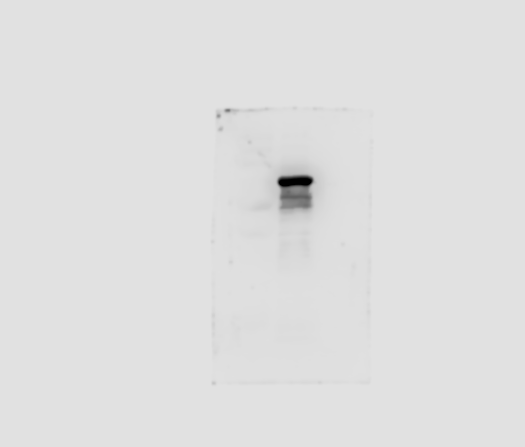

Supplement: Supplementary file 1 — Additional file 1. [file 12917_2022_3412_MOESM1_ESM.zip › H1 blot original image-2.tif]

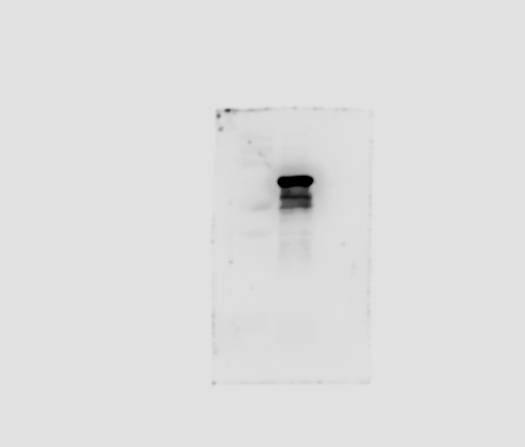

Supplement: Supplementary file 1 — Additional file 1. [file 12917_2022_3412_MOESM1_ESM.zip › H1 blot original image-3.tif]

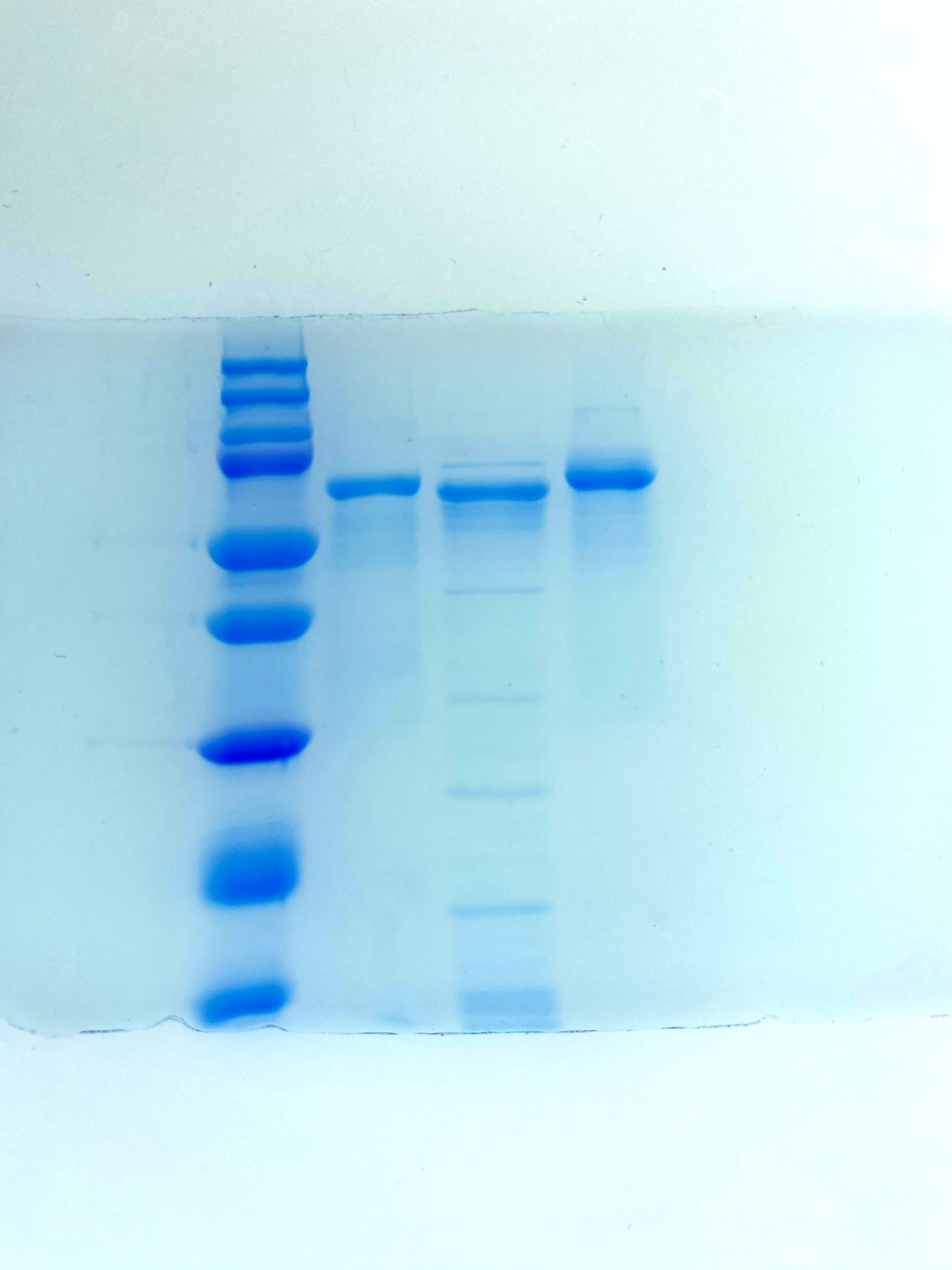

Supplement: Supplementary file 1 — Additional file 1. [file 12917_2022_3412_MOESM1_ESM.zip › H1719 SDS-PAGE original image.jpg]

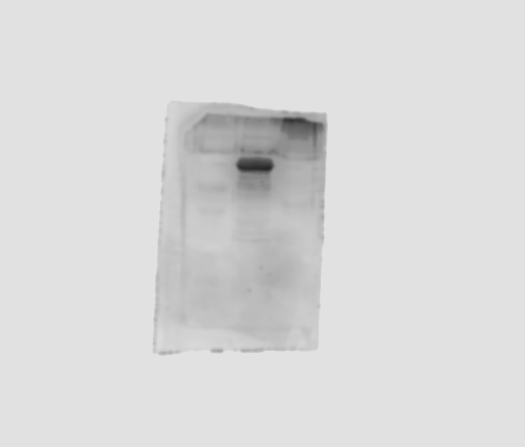

Supplement: Supplementary file 1 — Additional file 1. [file 12917_2022_3412_MOESM1_ESM.zip › H19 blot original image-1.tif]

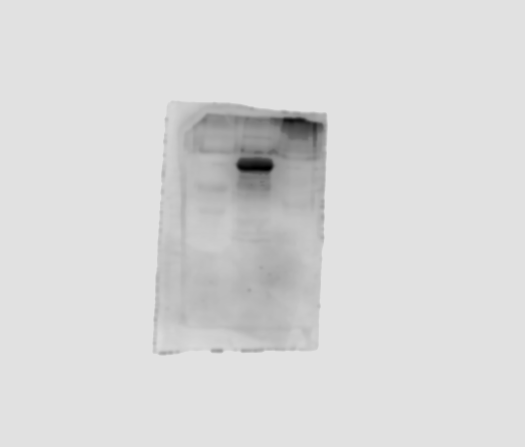

Supplement: Supplementary file 1 — Additional file 1. [file 12917_2022_3412_MOESM1_ESM.zip › H19 blot original image-2.tif]

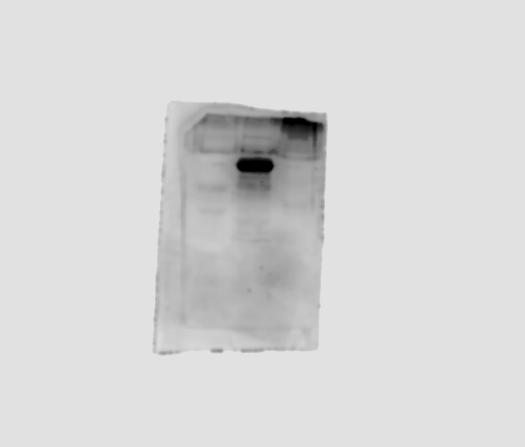

Supplement: Supplementary file 1 — Additional file 1. [file 12917_2022_3412_MOESM1_ESM.zip › H19 blot original image-3.tif]

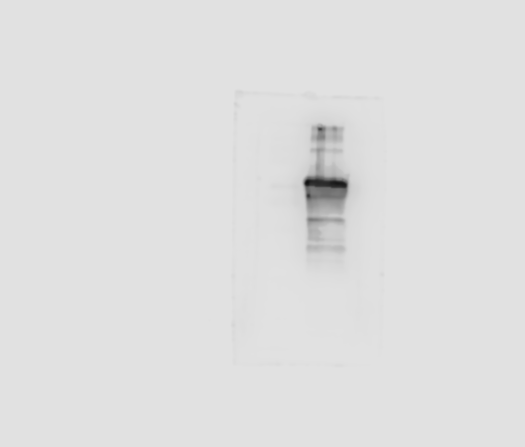

Supplement: Supplementary file 1 — Additional file 1. [file 12917_2022_3412_MOESM1_ESM.zip › H7 blot original image-1.tif]

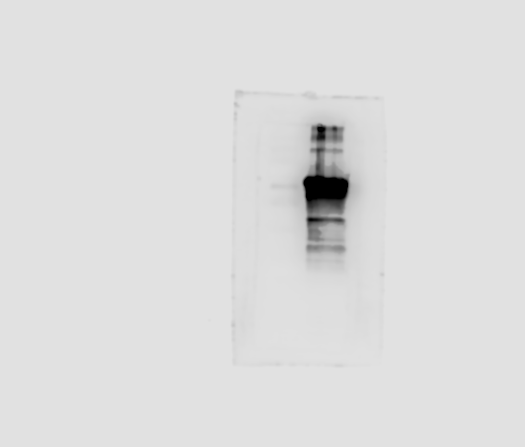

Supplement: Supplementary file 1 — Additional file 1. [file 12917_2022_3412_MOESM1_ESM.zip › H7 blot original image-3.tif]
